# Genome sequence and annotation of *Pyramidobacter* sp. strain YE332, isolated from a cattle rumen fermentation of Leucaena leaf

**DOI:** 10.1128/mra.01012-23

**Published:** 2024-03-19

**Authors:** Rosalind Gilbert, Carl Davis, Jenny Gravel, Diane Ouwerkerk

**Affiliations:** 1 Department of Agriculture and Fisheries, EcoSciences Precinct, Dutton Park, Queensland, Australia; 2 Queensland Alliance for Agriculture and Food Innovation, The University of Queensland, St Lucia, Queensland, Australia; Department of Biological Sciences, Wellesley College, Wellesley, Massachusetts, USA

**Keywords:** *Pyramidobacter*, rumen, Leucaena, fermentation, cattle

## Abstract

We report the 2.78-Mb circular genome sequence of *Pyramidobacter* sp. strain YE332, isolated from a fermentation of bovine rumen fluid, supplied with leaf material from *Leucaena leucocephala* cv. Cunningham. This genome sequence consists of 2,795,328 bp with 60% G + C content, 2,573 predicted coding DNA sequences, and 70 RNAs.

## ANNOUNCEMENT

The leguminous fodder shrub *Leucaena leucocephala* contains the non-protein amino acid mimosine, which is degraded by rumen microbes to 3-hydroxy-4-(1H)-pyridine (3,4-DHP), causing toxicity in cattle (1). Here, we report on *Pyramidobacter* sp. strain YE332, the most highly abundant Synergistota present within a 3.0-L continuous batch fermentation of bovine rumen fluid (Labfors 3, Infors HT), maintained under anaerobic conditions and supplied daily with 30 g Leucaena cv Cunningham leaf material (2). For isolation of strain YE332, anaerobic culture conditions were maintained at all times (3). An aliquot of fermenter fluid was spread on solid 20% rumen-fluid-based medium (4), with the addition of selective antibiotics (vancomycin 4 µg mL^−1^, neomycin 20 µg mL^−1^, colistin 5 mg mL^−1^, and erythromycin 40 µg mL^−1^), 2,3-DHP (250 mgL^−1^), mixed protein hydrolysate (0.5% wt/vol), and 10 mM glycine. Following incubation for 5 days at 39°C, a single colony was successively replica plated three times to ensure purity and transferred into broth of the same medium, and incubated at 39°C for 72 h, prior to cryopreservation.

High molecular weight genomic DNA was extracted from cells pelleted from broth cultures, using a cetyltrimethylammonium bromide protocol (5). PacBio RS II sequencing (P6-C4 chemistry on 1 flow cell) was undertaken on unsheared DNA using a 20-kb insert library with BluePippin size selection, resulting in 54,679 reads of N50 read length 19,441, which were assembled using SMRT portal software (v2.3.0; Pacific Biosciences) using default settings, with genome circularity and sequence overhang trimming undertaken using Berokka [v0.2.3 (6)]. This resulted in a complete genome (2,795,328 bp) consisting of a single circular contig and a second, shorter contig (2,777,081 and 18,247 bp). The genome was annotated by the NCBI Prokaryote Genome Annotation Pipeline [v6.6 (7)], which predicted 2,643 genes, including 2,573 coding DNA sequences, 70 RNAs (five 5S, four 16S, and four 23S), 54 tRNAs, 3 non-coding RNAs, and 6 CRISPR arrays.

Assignment of taxonomy based on the 16S rRNA gene sequence indicated this strain to be 99.97% identical to that of *Pyramidobacter* sp. strain C12-8 (8) and 99.84% identical to *Pyramidobacter* sp. strain CG50-2 (unpublished, GCF_003612005.1), which were isolated from the bovine and capris rumen, respectively. Whole-genome comparison with average nucleotide identity (9) and dDDH values [Genome-to-Genome Distance Calculator v3.0 (10, 11)] found strain YE332 to be 99.01% and 98.41%, and 91.30% and 85.40% identical to the *Pyramidobacter* sp. strains C12-8 (GCF_002007215.1) and CG50-2 (GCF_003612005.1), respectively.

Annotation of metabolic genes using EggNOG Mapper [Galaxy v2.1.8 (12, 13)] indicated that in accordance with other bacteria classified within the phylum Synergistota (14), strain YE332 had relatively few CAZy genes for carbohydrate metabolism, instead preferring the metabolism of proteins and amino acids (214 COG category E proteins encoded). This indicated that *Pyramidobacter* may be a secondary degrader, utilizing the by-products of microbial plant digestion released into the rumen liquor by primary fermentation of carbohydrates and microbial lysis. This genome sequence contributes new information to improve current understanding of the role of Synergistota and *Pyramidobacter* in the rumen microbial ecosystem.

## Data Availability

The whole-genome sequence for *Pyramidobacter* sp. strain YE332 has been deposited in GenBank under the accession number CP133038.1, BioProject number PRJNA1004043, and BioSample number SAMN36920621. The raw sequence reads are available in the Sequence Read Archive (SRA) database under accession number SRP459544.
